# Revealing the Micro-scale Signature of Endemic Zoonotic Disease Transmission in an African Urban Setting

**DOI:** 10.1371/journal.ppat.1005525

**Published:** 2016-04-08

**Authors:** Hervé Bourhy, Emmanuel Nakouné, Matthew Hall, Pierre Nouvellet, Anthony Lepelletier, Chiraz Talbi, Laurence Watier, Edward C. Holmes, Simon Cauchemez, Philippe Lemey, Christl A. Donnelly, Andrew Rambaut

**Affiliations:** 1 Institut Pasteur, Unit Lyssavirus Dynamics and Host Adaptation, WHO Collaborating Centre for Reference and Research on Rabies, Paris, France; 2 Institut Pasteur de Bangui, Bangui, République Centrafricaine; 3 Institute of Evolutionary Biology, University of Edinburgh, Ashworth Laboratories, Edinburgh, United Kingdom; 4 Department of Infectious Disease Epidemiology, Imperial College London, London, United Kingdom; 5 Medical Research Council Centre for Outbreak Analysis and Modelling, Department of Infectious Disease Epidemiology, Imperial College London, London, United Kingdom; 6 INSERM, UMR 1181 and Institut Pasteur, B2PHI, Paris, France; 7 Faculté de Médecine Paris Ile de France-Ouest, Université de Versailles–Saint-Quentin, Versailles, France; 8 Marie Bashir Institute for Infectious Diseases & Biosecurity, Charles Perkins Centre, School of Life and Environmental Sciences and Sydney Medical School, Sydney, Australia; 9 Mathematical Modelling of Infectious Diseases Unit, Institut Pasteur, Paris, France; 10 Rega Institute, KU Leuven, Leuven, Belgium; 11 Fogarty International Center, National Institutes of Health, Bethesda, Maryland, United States of America; Cornell University, UNITED STATES

## Abstract

The development of novel approaches that combine epidemiological and genomic data provides new opportunities to reveal the spatiotemporal dynamics of infectious diseases and determine the processes responsible for their spread and maintenance. Taking advantage of detailed epidemiological time series and viral sequence data from more than 20 years reported by the National Reference Centre for Rabies of Bangui, the capital city of Central African Republic, we used a combination of mathematical modeling and phylogenetic analysis to determine the spatiotemporal dynamics of rabies in domestic dogs as well as the frequency of extinction and introduction events in an African city. We show that although dog rabies virus (RABV) appears to be endemic in Bangui, its epidemiology is in fact shaped by the regular extinction of local chains of transmission coupled with the introduction of new lineages, generating successive waves of spread. Notably, the effective reproduction number during each wave was rarely above the critical value of 1, such that rabies is not self-sustaining in Bangui. In turn, this suggests that rabies at local geographic scales is driven by human-mediated dispersal of RABV among sparsely connected peri-urban and rural areas as opposed to dispersion in a relatively large homogenous urban dog population. This combined epidemiological and genomic approach enables development of a comprehensive framework for understanding disease persistence and informing control measures, indicating that control measures are probably best targeted towards areas neighbouring the city that appear as the source of frequent incursions seeding outbreaks in Bangui.

## Author Summary

Rabies is an endemic and zoonotic disease circulating in most African and Asian countries and is mainly transmitted by dogs. Despite its importance for public health, little is known about the spatiotemporal dynamics of rabies in dogs in an urban setting. Using spatial, temporal and virus genetic information, we reconstructed the fine-scale evolutionary and epidemiological dynamics of RABV among the dog population in a major African city—Bangui in the Central African Republic. Notably, we document that rabies is not self-sustaining in the city, and that numerous reintroductions of the virus occur in the city, generating a succession of epidemic waves. The data obtained here will inform the development of tailored control measures and avoid wasting resources, revealing that control measures are probably best targeted towards neighbouring geographic areas rather than to the city itself.

## Introduction

Understanding the processes responsible for the maintenance and extinction of infectious diseases in specific geographic locations is essential for the establishment of locally relevant control strategies. Resources for infectious disease surveillance are always in limited supply, especially those dedicated to zoonotic neglected tropical diseases [1]. As a consequence, a lack of comprehensive data on incidence and transmission chains often prevents the accurate inference of disease dynamics and patterns of transmission. Fortunately, the development of approaches combining both epidemiological and genomic data provides new opportunities to explore this problem at a fine scale offering a robust framework to account for the highly stochastic nature of both the disease transmission and observation processes.

Our focus is dog mediated rabies, one of the most neglected of all infectious diseases. Every year, nearly 15 million people receive post exposure prophylaxis and an estimated 59,000 people die from rabies [2], with 98% of these resulting from dog bites and with a burden disproportionately affecting poor rural communities and children. However, regional and national priorities for research and control are difficult to establish due to poor background data. A number of major challenges are apparent [3]. In particular, rabies surveillance systems are often non-existent or inadequate due to the lack of rabies awareness among health professionals and the target population. In addition, there is a lack of legislation for compulsory notification and an absence of laboratory confirmation leading to unreliable diagnoses. In addition, the long-term presence of rabies relies on the zoonotic source of transmission and domestic dog populations remain the principal target for prevention and interventions [4]. Evidently, uncertainty surrounding the transmission dynamics and maintenance of dog rabies must be addressed to plan, optimize and implement strategies. Intrinsic cycles and traveling waves linked to rabies-induced fluctuations in susceptible animal populations are observed in wild carnivores in Europe and North America [5,6]. In contrast, little is known about the spatial and temporal dynamics of rabies in domestic dogs at a large scale [7–10], and almost nothing at local scales, including the factors that lead to disease endemicity in an urban setting. Given the tight social and spatial structure of dog populations, the dynamics of rabies in dog populations are often considered in the context of meta-populations where endemic populations provide a source of infection for sink populations [11,12]. In such settings, targeted interventions would clearly benefit enormously from identifying these sources of infection.

Herein, we utilize a unique combination of epidemiological data and virus genome sequence data to determine the local population dynamics of a neglected tropical disease. We apply this method to determine the local population dynamics of rabies, particularly its rate of importation and transmission potential at a highly localized scale in Bangui, the capital city of Central African Republic (CAR), information that will be critical to its overall control.

## Results

### Periodicity of rabies in Bangui

Wavelet analysis and periodograms were performed on the data collected on dogs in Bangui from 1990 to 2012. Periods of 53.4 and 89.0 months were observed. No cases were detected over periods of 289 days, 470 days and 388 days, between 1996–1997, 2005–2006 and 2010–2012, respectively. Such long ‘epidemiologically silent’ intervals are intriguing and, given the mean incubation period plus infectious period of rabies in dogs (25.1 days) [7], suggest that the virus may not persist in the city between consecutive epidemiological periods.

### Phylogeny and time-scale of RABV evolution

**Fig 1:**
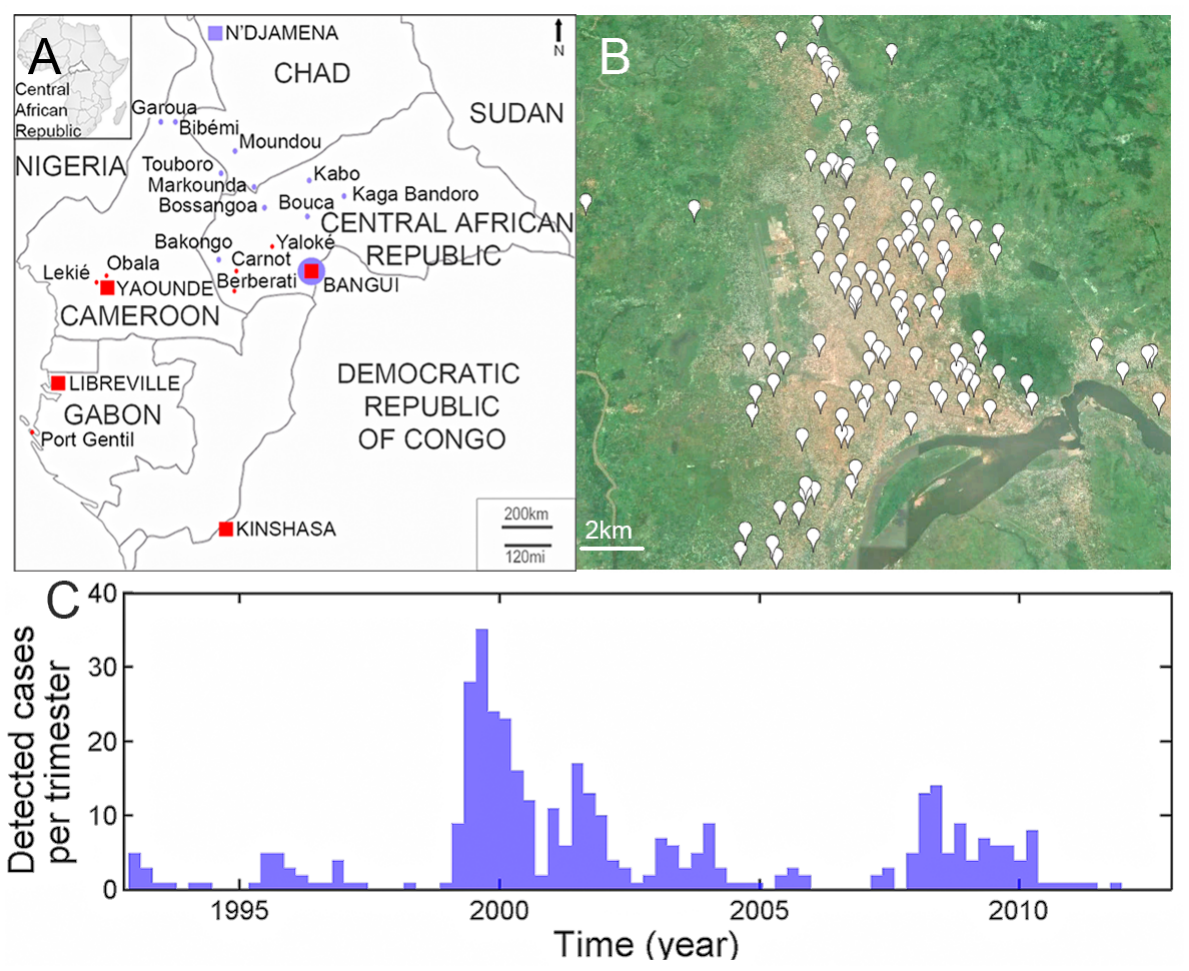
Sampling of RABV samples. Map of the Central African Republic and neighbouring countries (a) showing the location (red points and squares) of the 162 isolates of RABV analyzed in this study. The cities in red indicate the presence of RABV belonging to the Africa 1 clade. Cities in blue represent those where RABV belonging to the Africa 2 clade were found. In Bangui, both clades were identified. Detailed map of Bangui (b), showing the location of the 122 samples analyzed in the context of the landscape. Number of specimens positive for rabies (c) by trimester during sentinel surveillance, January 1993 to March 2012.

**Fig 2:**
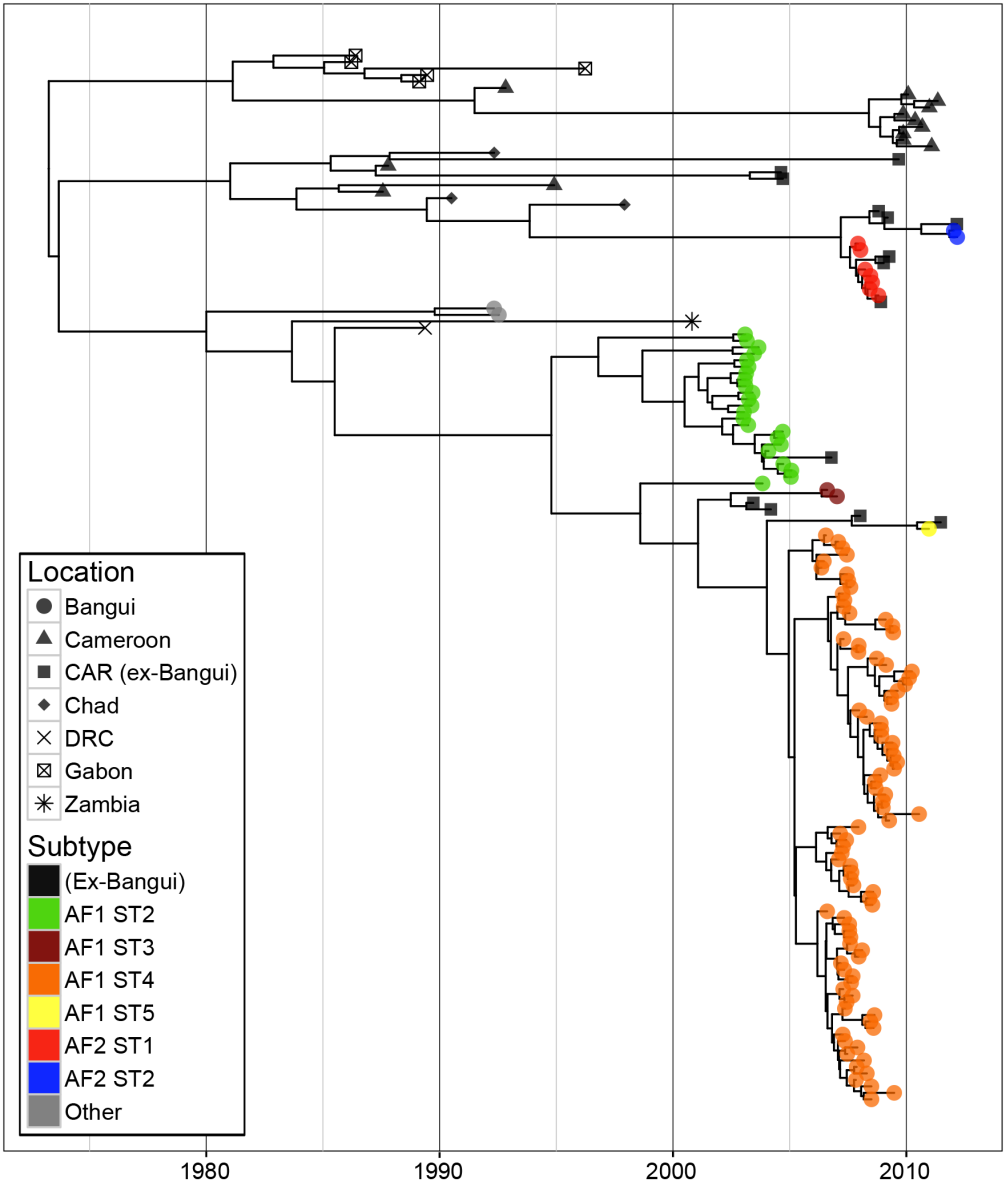
MCC tree of 162 sequences from the Central African Republic and other locations in Africa estimated from 5000 nt of dog RABV genome. Tips representing isolates from Bangui are coloured according to the selected subtypes of RABV; other tips are colored by location. Tip times are scaled to the date of sampling (years) and branches are estimated in time units as indicated by the time bar.

To further investigate the spatiotemporal dynamics of rabies in CAR, and to verify the likely periodic introduction of RABV in Bangui, rabies virus (RABV) isolates from Bangui collected from 2003 to 2012 were sequenced and analysed, together with those of viruses obtained earlier (in 1992) from Bangui and from neighboring or geographically close countries (Fig 1, Table A and Fig A in S1 Text). We first inferred the evolutionary history of rabies virus in this area using a set of 162 RABV sequences (5061 nt; encompassing the N, P, M, G and intergenic G-L region) (Fig 2). The tree topology is similar to earlier phylogenetic analyses, with sequences divided into two major clusters identified as the 'Africa 1' (AF1) clade which represents an African subset of the ‘Cosmopolitan’ clade, and an ‘Africa 2’ (AF2) clade [13–15]. Most of the isolates reported in Bangui belonged to the AF1 clade. Root-to-tip linear regression analysis of the 2003–2012 CAR sequences from those two clusters established that there was a strong temporal signal in these data, with correlation coefficients of 0.79 (AF1) and 0.71 (AF2) for the relationship between root-to-tip divergence and sampling date. We then estimated the time-scale of the evolutionary history of the 162 RABV isolates using a Bayesian Markov chain Monte Carlo approach [16]. The mean rate of nucleotide substitution estimated in this manner, assuming an uncorrelated lognormal relaxed molecular clock model, was 5.9x10^-4^ substitutions per site, per year (95% highest posterior density (HPD) interval, 4.4–7.5x10^-4^ subs/site/year). These rates are within the range of those previously estimated for the N and G genes in various dog RABV sample data sets in Africa [9,15] and worldwide [17].

### Evidence of several independent introductions of RABV into Bangui

Clades in the RABV phylogeny were regarded as representing likely new introductions into Bangui if it was possible to go back a time *L* from their root node without encountering either the start of sampling in 2003, or a node that could not be ruled out as representing a transmission to a dog that was sampled. *L* is intended to be a time period that is sufficiently long so that the probability was very low that the virus had been transmitted in Bangui, while sampling was going on, but without any dogs in the transmission chain being sampled. In addition, we made the *a priori* assumption that there were at least three introductions comprising AF2 and the two waves of AF1 (2003–2005 and 2006–2012). This was because surveillance only began in 2003, such that for larger values of *L* it was impossible to go back *L* years from the estimated times of the root nodes of AF2 or the second AF1 wave without encountering the start of sampling, and hence our algorithm alone could never determine that undetected passage for *L* years was unlikely. Not making this assumption was deemed overly conservative due to the absence of any detected clinical cases in the city for 15.5 months between the two waves. Our initial assumptions were therefore that there were at least three separate introductions, but for values of *L* of 1, 2, 3, 4 and 5 years the analysis identified 7, 6, 5, 5 and 5, respectively; even very conservative choices of *L* indicated more than were obvious from the epidemiological data. We designate these introductions as subtypes (ST): variants of RABV circulating during the time frame of the study.

**Fig 3:**
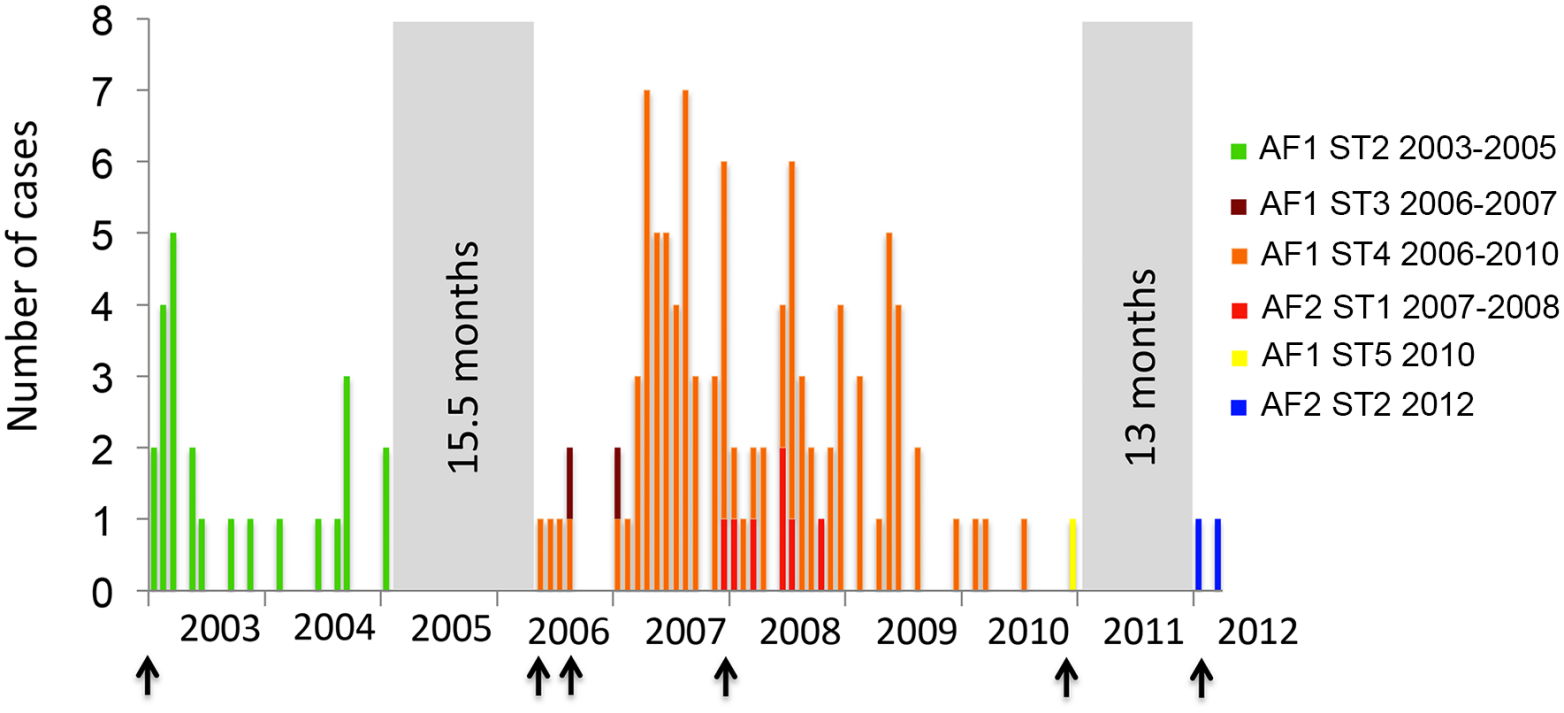
Temporal distribution of RABV subtypes in Bangui as determined by the MCC analysis (number of specimens positive for rabies by months from January 2003 to March 2012 and same color code as in Fig 2). Black arrows indicates RABV introduction events in the city (first introduction of a new subtype circulating in Bangui). Grey areas indicated periods without any reported cases.

To determine a plausible value of *L*, we used estimates of the assumed detection probability *ρ* (range 10%-50%) to account for low surveillance efforts and combined them with data concerning the incubation plus infectious period reported for dogs in Africa [8]. From this, we were able to estimate the probability of the presence of a ST in Bangui without any dog in the transmission chain being sampled. The expected number of dogs in the chain is 14.37 in one year and 28.89 in two years. The probability of not detecting one of these dogs up after one year is 0.22 for a 10% sampling fraction, 0.0405 for 20% and 4.71x10^-5^ for 50%. At two years, the probability is less than 5% (0.0477) even for a 10% value of *ρ*. A value for *L* of two years therefore seems reasonable, from which we can infer at least 6 separate introductions of RABV occurred in Bangui during the time course of this study (2003–2012) (Fig 3). Importantly, this estimate may be conservative because sampling only started in 2003, and the posterior median TMRCA of all the isolates collected in Bangui during 2003–2004 occurs during 1998, such that it was impossible to determine whether these isolates were the result of a single introduction. In addition, we cannot exclude that the same ST was repeatedly introduced into the city during the time course of the study. The six different introductions were due to 4 different STs of AF1 and 2 different STs of AF2. One viral subtype was always predominant during each of the waves but the reported ST shifted from one wave to another (Fig 3). AF1 ST1 was predominant in 2003–2005 while AF1 ST2 became predominant in 2006–2010. In 2012, another new ST appeared in Bangui (AF2 ST2). Our findings are therefore consistent with a series of distinct clusters of RABV originating from independent introduction into Bangui, each of which then experienced relatively limited transmission in the city.

### Phylogeography of RABV in Bangui

To determine the spread of rabies virus in the city, we used a combination of phylogenetic and coalescent approaches to connect the historical process of dog rabies virus evolution in Bangui together with epidemiological parameters. Our aim was to integrate spatial dynamics with temporal inferences to follow the movement of RABV, and hence infected dogs, in the city. A Mantel r-test was performed on the pairwise genetic differences versus physical distances of the samples, giving a significant (p = 0.023) but weak (r = 0.074) positive association between the two differences. A continuous traits phylogeographic analysis was then performed in BEAST, using the geographical coordinates of the largest data set of samples corresponding to AF1 ST4. The analysis of how the infected areas changes with time indicated that these viruses initially arrived from the Southwest and then eventually dispersed to the centre of the city (Fig B1 in S1 Text). The virus then largely spread along a Southeast Northwest axis in the city (Fig B2 in S1 Text). The estimated diffusion rate for the AF1 ST4 strain in Bangui obtained from the data was 0.9 km per year (95% HPD interval: 0.65–1.2 km)

### Temporal trends and role of importation of rabid dogs into Bangui

**Fig 4:**
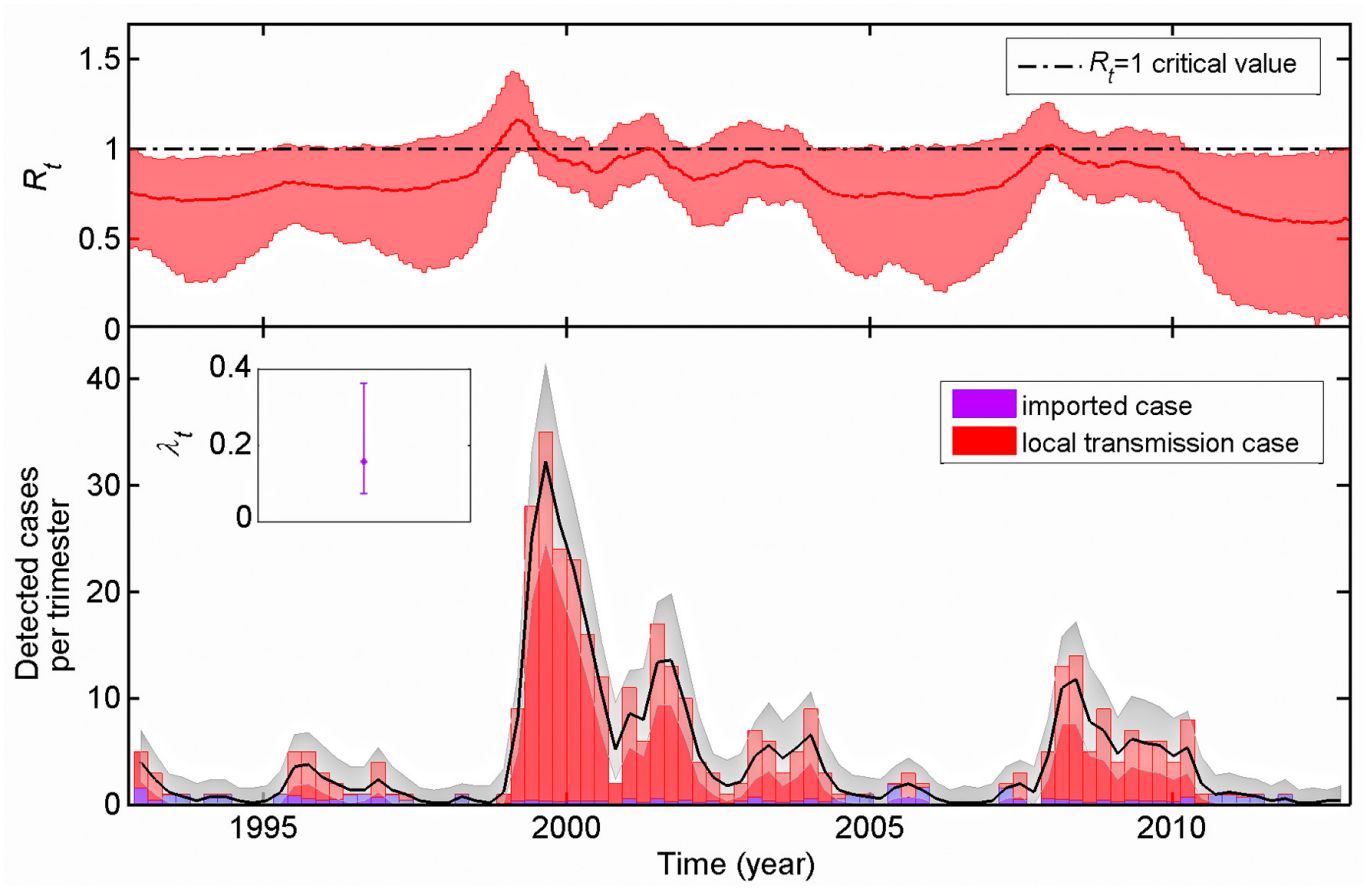
Estimation of the rate of introduction of rabid dogs in Bangui, *λ*_*t*_ (in rabid dogs imported to Bangui per week), the instantaneous effective reproduction number, *R*_*t*_, and observed number of rabid dogs infected locally (red bar) or from outside the city (blue bar) and simulated number of rabid dogs from the model (black line: posterior median; grey area: 95% credible interval of the posterior distribution). If *R*_*t*_, is above 1, a local self-sustaining epidemic in dogs may occur in the city.

To determine the population dynamics of rabies in Bangui, the instantaneous effective reproduction number *R*_*t*_ and the rate of introduction *λ*_*t*_ were estimated based on the incident cases from 1990 to 2012. Strikingly, we observed relatively little variation in *R*_*t*_ over time, with median monthly point estimates in the range 0.8 to 1.3 (Fig 4, Fig C in S1 Text), with *R*_*t*_ rarely significantly above the critical value of 1 (only during 12 weeks in 2009 according to our best model). Substantial temporal variation in the 95% credible intervals for *R*_*t*_ was observed and likely reflects variation in the number of reported incident cases rather than dramatic changes in the underlying transmission dynamics of the disease, i.e. since *R*_*t*_ describes infectiousness of a case, the number of incident cases in a time period determines the precision of the estimate [18]. The best model (Table B in S1 Text) assumed a constant exogenous rate of introduction *λ*_*t*_ throughout the study period, with a median estimate of 0.13 (95% credible interval CI: 0.07, 0.27) rabid dogs imported to Bangui per week (i.e. ~7 per year) (Fig C, D and E in S1 Text). Given the posterior distribution of *R*_*t*_ and *λ*_*t*_, the simulated number of rabid dogs from the model was in good agreement with the observed counts (Fig 4c). Based on the estimated parameters, we were able to infer the likely proportion of dogs that became rabid as a result of a local transmission event or were imported cases. Accordingly, from 1992 until 2012 we inferred that among 390 rabid dogs observed, approximately 10% of them (~40) were introduced in Bangui (blue bars in Fig 4, lower panel). The sensitivity analysis indicated that the inferred pattern of temporal variation in both *R*_*t*_ and *λ*_*t*_ was robust to changes in the assumed detection probability (Table C in S1 Text). Given an estimated median for *R*_*t*_ of 0.84 over the whole study period, an outbreak seeded by a single case would end, on average, after a total of 6.3 cases (including the index case) with a variance linked to heterogeneity of transmission [19,20]. While our analysis does not allow us to directly reconstruct the chains of infection, given estimated values for the median *R*_*t*_ and *λ*_*t*_, (and assuming a Poisson distribution of cluster sizes, i.e. no heterogeneity in infectiousness), we expect the occurrence of large clusters; for instance, over the whole study period we expect 3.2 clusters larger than 50 cases (10 observed cases given the 20% best inferred sampling detection). Finally, between 2003 and 2012, our statistical analysis predicted that 10 observed cases had a probability greater than 90% of having been imported. In contrast, the genetic analysis allowed us to determine that 6 new virus subtypes were introduced during the same time period. Four importations (in late 2003, early 2006, late 2010 and early 2012) were identified by both methods. The known importations (from genetic information) from mid-2006 and late 2007 were missed by the statistical analysis, although not surprisingly as they arose during a large infection cluster. Finally, 6 likely imported cases were missed by the genetic analysis, most likely reflecting multiple importations of viruses with very similar sequences. In sum, our analyses confirms that, although rabies is almost endemic in Bangui, its dynamics are in fact driven by a high rate of importation and characterized by frequent cluster die-out.

## Discussion

Phylogenetic and coalescent-based methods allow the explicit use of spatial information within a statistical framework [9,21,22]. When coupled with mathematical models for estimating time-varying reproduction numbers during epidemics this permits practical and operational questions to be asked. In the case of neglected and re-emerging zoonoses such as rabies, it is particularly important to understand the details of transmission dynamics to design efficient and sustainable strategies for dog rabies control and elimination. Here, we described transmission patterns of RABV infection in a dog population in Bangui, a relatively large African city with nearly 900,000 inhabitants. In this city, the domestic dog is considered to be the only reservoir of rabies virus—that is, the only set of epidemiologically connected animal populations maintaining rabies infection in a defined geographical area [23]–although actions taken to control the disease in dog populations are limited.

Our study demonstrates that the dog population of Bangui experiences the extinction of RABV transmission chains separating small outbreaks and that reflects a succession of epidemic waves, a pattern that is consistent with previous observations in Africa [7,8]. Importantly, when examined over the long term this gives the false impression of a stable endemic infection. Indeed, we noted that the instantaneous effective reproduction number *R*_*t*_ was rarely significantly above the critical value of 1. Wide temporal variation in credible intervals for *R*_*t*_ were observed, but are likely to reflect more the variation in incident cases reported rather than changes in the dynamics of the disease per se [18]. These estimates of *R*_*t*_ are surprisingly low with median monthly point estimates (0.8 to 1.3) spanning the range of lower estimates of the basic reproduction number, *R*_0_ (not instantaneous), obtained from compiled outbreak data from other localities [8]. These estimates of *R*_*t*_ are consistent, although often lower than the *R*_0_ estimated (*R*_0_ = 1.1) for another African city, Nd'jamena in Chad [10]. It is interesting to note that the periods that correspond to clear outbreaks have *R*_*t*_ above 1 (presumably corresponding to beginning of AF1 ST2 and of AF1 ST4) and that during the exponential growth of these outbreaks, *R*_*t*_ corresponds to *R*_0_. Although *R*_*t*_ rarely exceeds 1, this is consistent with other endemic persistent infections (e.g. see variation in *R*_*t*_ for polio, [24]).

The inference of successive small transmission chains is supported by our phylogenetic analysis that reveals the existence of repeated introductions of RABV from outside the city. Notably, the subtype of reported RABV circulating in the city shifted from one wave to the next, indicating a sudden occurrence and rapid disappearance for most of the ST identified. This periodic replacement of the subtypes argues against persistent diffusion of RABV through the local movement of rabid dogs in the city. Some of the subtypes infecting dogs in Bangui belonged to the Africa 2 clade, which was originally only found in the northern part of the country. Hence, this confirms the long-distance spread of RABV (several hundreds of km) likely associated with human-mediated displacement of dogs infected with rabies [9], and its role in repeated and successful re-introduction of RABV in rabies-free areas. In turn, our mathematical model supports a constant exogenous rate of introduction *λ*_*t*_ throughout the study period with a median estimate at 0.13 (95% CrI: 0.07, 0.27) rabid dog imported to Bangui per week. This was substantiated by the phylogenetic analysis which allowed us to estimate that at least 6 different ST were imported in the city between 2003 and 2012. Further, the movement of infected dogs in the city as suggested by the rate of diffusion of one RABV ST in the city (0.9km/year) from the phylogeographic analysis appeared relatively low compared to other studies performed at a larger scale in Africa [8,9], indicating that the spatial spread of RABV within the urban dog population is limited. In sum, both the epidemiological and genomic data paint a consistent picture of serial importation of distinct viral clusters that experience relatively little evolution in the city.

Given that epidemiological surveillance of rabies in animals is not exhaustive, a substantial proportion of animal cases were likely never detected and therefore did not appear in our data set, in turn leading to an underestimation of the burden of rabies. However, this absence of information did not interfere with the reliability of the conclusions obtained, as these potential sampling biases were taken into account during the analyses. Further, estimates of *R*_*t*_ over time are generally robust to underreporting as long as the proportion of underreported cases is constant over time. While assuming constant reporting was reasonable from field experience, it is likely that some variation occurred, particularly that reporting increased during periods of higher incidence as surveillance became more active. If such variations in reporting occurred, our estimate of *R*_*t*_ during period of high incidence would be slightly overestimated, as the increase surveillance would reveal cases that would otherwise not be detected. However, we predict that any possible reporting bias would have a minimal impact on our estimate of importation as it relies more on period of low incidence. Together, our central conclusions (i.e. of disease dynamics driven by a high rate of importation and characterized by frequent cluster die-out) appear to be robust, and even conservative, to increased reporting during periods of high incidence.

The overall consistency of the results obtained by our two approaches, mathematical and phylogenetic modeling, is therefore reassuring and complements other approaches combining genetic and epidemiological data enabling the reconstruction of partially observed transmission trees and estimating the number of cases missing [25–29]. Further, our approach is designed to incorporate an exogenous force of infection and can be applicable in a wide range of settings including those where handling missing data is critical. We still have to determine how best to integrate commonly collected data types (incidence data, time series of reported cases and molecular sequence, etc.) to inform pathogen persistence and control. However, we have shown here that the combined use of mathematical and phylogenetic approaches has provided a new understanding of the transmission of RABV. This information could be further used to inform RABV prevention and response policies in defined areas in a way that maximizes cost-effectiveness and operational efficiency [1,30]. For example, it would be useful to identify areas where surveillance is critical and low reporting rates might leave epidemics undetected, and would aid the rapid implementation of public health strategies targeting identified geographical regions at risks.

Drawing upon these epidemiological and evolutionary data allows us to address more fundamental questions relating to the persistence of rabies and to the role of different dog populations. The size and structure of domestic dog populations in space and time can be extremely variable and difficult to measure, but in general can be thought of as a spatial mosaic of large and small populations that are occasionally connected. The respective roles of small metapopulation-structured versus large and more homogeneous dog populations in the maintenance of a zoonotic disease such as rabies are unknown [1]. The coupling of large and small dog populations and their spatial connectivity may therefore be the critical feature that generates waves and persistence of RABV at a regional scale. As a consequence, the complex process of persistence seems therefore to operate on multiple scales among which the metapopulation structure of dogs could play a major role compared to densities [1,7,8,31]. The spatial distribution and size of subpopulations can cause spatial hierarchies of metapopulation dynamics or travelling waves of infection [32]. It is therefore essential to identify sub-populations important in source-sink dynamics or bottleneck areas, each requiring tailored management plans [27,30]. More generally, our data demonstrate the need to understand hierarchical ('core-satellite') epidemic dynamics for the control of established and re-emerging diseases [23,33]. The exploration of the potential ecological and epidemiological drivers behind these findings is a major challenge for future research. From this perspective, the collection and study of rabies specimens collected at a larger geographical scale including peri-urban and rural areas would be valuable. The role of metapopulations and in particular of the minimum viable metapopulation (MVM) size has also been addressed in rabies [34] and in other systems with short infectious period such as measles, in which large focal communities have been proposed to act as refuges that allow metapopulation persistence [35,36].

Phylogenetic and coalescent-based methods coupled with mathematical models also allow us to address practical and operational questions regarding rabies control, particularly the optimal strategies for vaccination and allocations in situations where resources are limited. The dynamics of rabies and the effectiveness of vaccination for reducing the occurrence of rabies are strongly influenced by the spatial distribution and the metapopulation structure of dog population [11,12]. According to our study, control measures are best directed toward preventing spill-over from neighboring populations and the introduction of novel RABVs into this target dog population, rather than only targeting transmission within the urban dog populations.

## Materials and Methods

### Epidemiological data collection and selection

The State health department of CAR conducted all observations and euthanization (if necessary) of animals suspected of rabies in Bangui, according to established national standardized protocols and WHO guidelines [37]. The Institut Pasteur of Bangui is the only laboratory in the country that is designated by the Ministry of Health of CAR to conduct the collection of specimens from dead animals suspected of rabies and to perform rabies laboratory diagnosis [38]. Rabies diagnosis was performed by direct immunofluorescence on smears of suspected animals, and confirmed in parallel using RT-PCR as previously described [37,39]. Confirmed animal cases from CAR were collated over the period 1990–2012. The data collected include the date of death, the species, the animal’s city of origin and starting from 2003 the spatial coordinates for all the isolates originating from Bangui (Table A in S1 Text).

### Estimation of temporal trends of rabies

To determine periodic components of the monthly case time series, we performed a spectral analysis [40] on data collected from 1990 to 2012. We then used the number of dogs reported as rabid in Bangui and their date of submission to rabies diagnosis from 1992 to 2012 to infer the dynamics of rabies transmission. The instantaneous effective reproduction number *R*_*t*_ is the average number of dogs that a single dog infected at time *t* could be expected to infect should conditions remain unchanged. Simple methods were developed to estimate trends in *R*_*t*_ from the epidemic curve of incident cases in the context of closed epidemics [18,41]. Here, we expanded these approaches for the situation where there is an exogenous force of infection. The number of incident cases at time *t*, *I*_*t*_, was therefore modelled as a Poisson process with mean $\lambda_{t}+R_{t}\sum_{s=1}^{t}I_{t-s}w_{s}$, where *λ*_*t*_ was the rate of introduction of rabies into the city and *w*_*S*_ was the distribution of the serial interval. The serial time interval, accounting for the incubation and infectious periods, was assumed to be constant over time and to follow a Gamma distribution parameterised using data presented in Hampson *et al*. [8] with a mean serial interval of 25 days and a variance of 171 days^2^. Further, the number of observed rabid dogs at time *t* followed a binomial distribution *Bin*(*I*_*t*_,*ρ*), where the detection probability *ρ* was assumed to be constant over time. Given the data, the unit time of the inference model was the week; thus data on incidence were aggregated weekly (e.g. *I*_*t*_ corresponds to the number of incident case during week *t*). Both *R*_*t*_ and *λ*_*t*_ were initially allowed to vary according to random walks characterised by their respective variances parameters ($\sigma_{R_{t}}^{2}$ and $\sigma_{\lambda_{t}}^{2}$) (see [42, 43, 44] for details). Specifically, the value *R*_*t*_ was modified using *R*_*t*_ = *R*_t−τ_+*ε* with *ε* a random variable following a normal distribution with mean zero and variance $\sigma_{R_{t}}^{2}$ and *τ* the time window at which *R*_*t*_ was assumed to change (the variance $\sigma_{R_{t}}^{2}$ was increased during the 3 largest outbreaks to allow larger volatility in *R*_*t*_, see Fig C, D and E in S1 Text). The same procedure was applied to *λ*_*t*_. Given the limited data available, we explored the monthly variation of *R*_*t*_ (*τ* = 1 month) and 3 models for *λ*_*t*_: i) a constant rate of rabies introduction throughout the period (*λ*_*t*_ = *λ*), ii) quarterly variation in *λ*_*t*_ (*τ* = 3 month), to explore potential seasonal trends and iii) annual variation in *λ*_*t*_ (τ = 12 month) to explore longer term trends. We estimated temporal trends in *R*_*t*_ and *λ*_*t*_ using a particle Monte Carlo Markov chain [42]. We report the posterior medians and 95% credible intervals of parameters. For each iteration of Markov chain we could extract a particular trajectory for the incidence (drawn randomly from the set of particles filtered at the end of the time series). The set of trajectories obtained constitutes the posterior incidence simulated. As all models had the same number of parameters, we compared them using an estimate of the mean deviance. A sensitivity analysis was performed to assess robustness of estimates to changes in the assumed detection probability *ρ* (range 10%-50%). To further evaluate and validate our model, we linked our results to branching theory that gives predictions about the cluster size due to single introduction, i.e. the total of number of cases (including the index case) after an outbreak is seeded by a single case and end [45]. Finally, for each day ‘t’, given estimation of *R*_*t*_, *λ*_*t*_ and the simulated trajectories, we could derive the force of infection due to either local transmission ($R_{t}\sum_{s=1}^{t}I_{t-s}w_{s}$) or imported cases (*λ*_*t*_). This allowed us to determine the probability that an observed case was imported rather than due to a local transmission event, and we analyzed those results in relation to the available genetic information of introduction of new virus subtypes.

### Bayesian evolutionary analysis of spatiotemporal RABV dynamics

A total of 162 RABV isolates were analyzed from which 139 originated from CAR, including 123 isolates collected during the period 2003–2012, 2 in 1992 from Bangui, and 14 from other cities in CAR (Fig 1, Table A in S1 Text). To complete the data set, sequences of 25 isolates from neighboring countries (Democratic Republic of the Congo, Chad, Cameroon, Gabon and Zambia) were also determined. Total RNA from the original brain samples (when available) was extracted using Trizol reagent (Invitrogen) according to the manufacturer’s instructions. To perform a reliable evolutionary analysis of dog RABV circulating in Bangui, we sequenced a large genomic area (42% of the viral genome) encompassing the N, P, M, G and intergenic G-L region, corresponding to positions 161 to 5221 of the RABV genome [46]. RNA extractions, RT-PCR and sequencing were performed as described previously [9,14,15,17] and with additional primers (Table D in S1 Text). All 162 sequences were aligned using the MUSCLE [47] plugin for Geneious 5.6.6 (Biomatters). Unrooted maximum likelihood phylogenetic trees for the entire 162-sequence alignment and for the AF1 and AF2 clusters in the CAR data were estimated using RAxML [48] employing 100 bootstrap replicates and the GTR+GAMMA nucleotide substitution model. The AF1 and AF2 phylogenies were used to investigate the clock-like behaviour in the data using the Path-O-Gen program, which maximises the Pearson product-moment correlation coefficient between sampling date and root-to-tip divergence for each isolate. Time-scaled phylogenetic trees were estimated using BEAST v1.7.5 [16] using the SRD06 nucleotide substitution model [49,50], an uncorrelated relaxed molecular clock [51] and a GMRF Bayesian skyride tree prior [52]. The analysis used multiple independent chains of 100 million MCMC states each, with samples taken sampling every 10,000 states. The convergence of parameter estimations was observed in all cases. The posterior set of phylogenetic trees from BEAST was used to identify separate introductions of RABV to Bangui; for full details see S1 Text. This procedure involves two numerical parameters, *L* and *P*. *L* is the length of time, in years, which is long enough that if rabies were being transmitted down a single chain while the population of infected dogs in the city was being sampled (with probability less than 1) upon death, we would expect to take at least one sample from a dog in the chain. *P* is a conservative estimate of the time from infection to death of an infected dog. In determining plausible values for *L* and *P*, we used previously-estimated figures for the distribution of incubation and infection periods of RABV in dogs [8] and the Welch-Satterthwaite approximation for the distribution of the sum of gamma-distributed random variables to estimate the distribution of the time from infection to death. The 99^th^ percentile of this distribution corresponded to 98.5 days, and we used this value for *P*. We also used this distribution to calculate the probability of sampling no dogs in a transmission chain for various values of *L* and sampling proportions.

To analyse the diffusion of RABV in the city of Bangui, a phylogeographic analysis of the 88 sequences of AF1 ST4 (i.e. the most common ST) was then conducted, also in BEAST, using the same nucleotide substitution model and tree prior as described above, along with a continuous spatial diffusion model [22]. This analysis utilizes the latitude and longitude at which each virus sample was taken, and uses them, in combination with the phylogeny, to estimate the parameters of a process by which viral lineages diffuse across the landscape and infer the locations of internal nodes. Branch-specific variation in diffusion rates was assumed to be lognormally-distributed. The prior distribution on the precision matrix of the diffusion process was the Wishart distribution described in [22] and an Exp(3) distribution was used for the standard deviation of the lognormal distribution modelling branch-specific diffusion rates. The SPREAD utility [53] was used to identify regions of Bangui with a 95% posterior probability for the presence of viral lineages, at different points in time, and these were visualised using Google Earth.

## Supporting Information

S1 Text Supporting information legends. Table A: Relevant epidemiological information for all RABV isolates analyzed in this study. Text S1: Bayesian evolutionary analysis of spatiotemporal RABV dynamics. Table B: Summary of the fit for models where *R*_*t*_ varied monthly while *λ*_*t*_ was either kept constant varied every 3 months or yearly. Table C: Sensitivity analysis on the assumption on the surveillance effort *ρ*. Table D: List of primers used in this study. Figure A: Detailed figure showing the MCC tree of 162 sequences from the Central African Republic and other locations in Africa estimated from 5000 nt of dog RABV genome with names of the tips. Tips representing isolates from Bangui are coloured according to the selected subtypes of RABV; other tips are colored by location. Tip times are scaled to the date of sampling (years) and branches are estimated in time units as indicated by the time bar. Posterior clade probability values (>0.9) are shown for key nodes. Figure B: Diffusion of RABV in Bangui. Figure B1: 80% highest posterior density (HPD) regions (red areas) for areas of Bangui where the AF1 ST4 strain of RABV was present, at different times during the epidemic, taken from the phylogeographical analysis. Figure B2: The maximum clade credibility tree for the ST1 AF4 RABV sequences (2007–2010), superimposed on a map of Bangui. Figure C: Fitting results with quarterly variation of *λ*_*t*_. Figure D: Fitting results with yearly variation of *λ*_*t*_. Figure E: Fitting results with constant importation *λ*_*t*_.
(DOCX)
