# Supplementary material for: Revealing the Micro-scale Signature of Endemic Zoonotic Disease Transmission in an African Urban Setting
Source: PLoS Pathog. 2016 Apr 8;12(4):e1005525. doi: 10.1371/journal.ppat.1005525 (PMC4825935; doi:10.1371/journal.ppat.1005525)
Supplement: S1 Text — Table A: Relevant epidemiological information for all RABV isolates analyzed in this study. Text S1: Bayesian evolutionary analysis of spatiotemporal RABV dynamics. Table B: Summary of the fit for models where R t varied monthly while λ t was either kept constant varied every 3 months or yearly. Table C: Sensitivity analysis on the assumption on the surveillance effort ρ. Table D: List of primers used in this study. Figure A: Detailed figure showing the MCC tree of 162 sequences from the Central African Republic and other locations in Africa estimated from 5000 nt of dog RABV genome with names of the tips. Tips representing isolates from Bangui are coloured according to the selected subtypes of RABV; other tips are colored by location. Tip times are scaled to the date of sampling (years) and branches are estimated in time units as indicated by the time bar. Posterior clade probability values (>0.9) are shown for key nodes. Figure B: Diffusion of RABV in Bangui. Figure B1: 80% highest posterior density (HPD) regions (red areas) for areas of Bangui where the AF1 ST4 strain of RABV was present, at different times during the epidemic, taken from the phylogeographical analysis. Figure B2: The maximum clade credibility tree for the ST1 AF4 RABV sequences (2007–2010), superimposed on a map of Bangui. Figure C: Fitting results with quarterly variation of λ t. Figure D: Fitting results with yearly variation of λ t. Figure E: Fitting results with constant importation λ t. (DOCX) [file ppat.1005525.s001.docx]

**Electronic supplementary material:**

Revealing the micro-scale signature of endemic zoonotic disease transmission in an African urban setting

Hervé Bourhy^1*^, Emmanuel Nakouné^2^, Matthew Hall^3,4^, Pierre Nouvellet^4,5^, Anthony Lepelletier^1^, Chiraz Talbi^1^, Laurence Watier^6,7^, Edward C. Holmes^8^, Simon Cauchemez^4,5,9^, Philippe Lemey^10^, Christl A. Donnelly^4,5^, Andrew Rambaut^3,11^

^1^Institut Pasteur, Unit Lyssavirus Dynamics and Host Adaptation, WHO Collaborating Centre for Reference and Research on Rabies, 75724 Paris cedex 15, France .

^2^Institut Pasteur de Bangui, Bangui, République Centrafricaine.

^3^Institute of Evolutionary Biology, University of Edinburgh, Ashworth Laboratories,

Edinburgh, UK.

^4^Department of Infectious Disease Epidemiology, Imperial College London, London, UK.

^5^Medical Research Council Centre for Outbreak Analysis and Modelling, Department of Infectious Disease Epidemiology, Imperial College London, London, UK.

^6^INSERM, UMR 1181 and Institut Pasteur, B2PHI, Paris, France.

^7^Faculté de Médecine Paris Ile de France-Ouest, Université de Versailles–Saint-Quentin, Versailles, France.

^8^Marie Bashir Institute for Infectious Diseases & Biosecurity, Charles Perkins Centre, School of Life and Environmental Sciences and Sydney Medical School, Sydney, Australia.

^9^ Mathematical Modelling of Infectious Diseases Unit, Institut Pasteur, Paris, France.

^10^ Rega Institute, KU Leuven, Minderbroedersstraat 10, 3000 Leuven, Belgium.

^11^ Fogarty International Center, National Institutes of Health, Bethesda, Maryland, USA.

**Table A: Relevant epidemiological information for all RABV isolates analyzed in this study.**

**Abbreviations: CAR: Central African Republic.**

| Seq ID | Access Number | Collection-date | Isolate | Country | Genotype | Lat-Lon |
| --- | --- | --- | --- | --- | --- | --- |
| 07128RCA_2003_016 | KT119634 | 06-Jan-2003 | Dog | CAR:Bangui | AF1 | 4.3630667 N 18.5872167 E |
| 07129RCA_2003_044 | KT119635 | 16-Jan-2003 | Dog | CAR:Bangui | AF1 | 4.33515 N 18.5309833 E |
| 07130RCA_2003_088 | KT119636 | 01-Feb-2003 | Dog | CAR:Bangui | AF1 | 4.3843528 N 18.5637056 E |
| 07131RCA_2003_090 | KT119637 | 02-Feb-2003 | Dog | CAR:Bangui | AF1 | 4.4007806 N 18.55875 E |
| 07132RCA_2003_112 | KT119638 | 10-Feb-2003 | Dog | CAR:Bangui | AF1 | 4.4100833 N 18.56745 E |
| 07133RCA_2003_140 | KT119639 | 20-Feb-2003 | Dog | CAR:Bangui | AF1 | 4.4257556 N 18.5302556 E |
| 07134RCA_2003_170 | KT119640 | 03-Mar-2003 | Dog | CAR:Bangui | AF1 | 4.37215 N 18.5469333 E |
| 07135RCA_2003_170 | KT119641 | 03-Mar-2003 | Dog | CAR:Bangui | AF1 | 4.4184667 N 18.5557 E |
| 07136RCA_2003_227 | KT119642 | 24-Mar-2003 | Dog | CAR:Bangui | AF1 | 4.43105 N 18.5465222 E |
| 07137RCA_2003_246 | KT119643 | 31-Mar-2003 | Dog | CAR:Bangui | AF1 | 4.3760778 N 18.5343333 E |
| 07138RCA_2003_283 | KT119644 | 28-Mar-2003 | Dog | CAR:Bangui | AF1 | 4.36955 N 18.57915 E |
| 07139RCA_2003_369 | KT119645 | 15-May-2003 | Dog | CAR:Bangui | AF1 | 4.3977833 N 18.5333167 E |
| 07140RCA_2003_411 | KT119646 | 30-May-2003 | Dog | CAR:Bangui | AF1 | 4.3881667 N 18.5547 E |
| othercity_07141RCA_2003_526 | KT119647 | 11-Jun-2003 | Dog | CAR:Berberati | AF1 |  |
| 07142RCA_2003_482 | KT119648 | 25-Jun-2003 | Dog | CAR:Bangui | AF1 | 4.3890139 N 18.5642889 E |
| 07143RCA_2003_671 | KT119649 | 02-Sep-2003 | Dog | CAR:Bangui | AF1 | 4.4237833 N 18.5404 E |
| 07144RCA_2003_841 | KT119650 | 03-Nov-2003 | Dog | CAR:Bangui | AF1 | 4.4223 N 18.53425 E |
| 07145RCA_2004_096 | KT119651 | 04-Feb-2004 | Dog | CAR:Bangui | AF1 | 4.3759 N 18.6174333 E |
| othercity_07146RCA_2004_197 | KT119652 | 12-Mar-2004 | Dog | CAR:Berberati | AF1 |  |
| 07147RCA_2004_426 | KT119653 | 24-Jun-2004 | Dog | CAR:Bangui | AF1 | 4.3872167 N 18.5430833 E |
| 07148RCA_2004_626 | KT119654 | 16-Aug-2004 | Dog | CAR:Bangui | AF1 | 4.4091389 N 18.5559556 E |
| 07150RCA_2004_705 | KT119655 | 14-Sep-2004 | Dog | CAR:Bangui | AF1 | 4.3931389 N 18.547 E |
| 07152RCA_2004_724 | KT119656 | 21-Sep-2004 | Dog | CAR:Bangui | AF1 | 4.4069611 N 18.5735611 E |
| 07154RCA_2005_052 | KT119657 | 19-Jan-2005 | Dog | CAR:Bangui | AF1 | 4.4025667 N 18.55405 E |
| 07155RCA_2005_074 | KT119658 | 27-Jan-2005 | Dog | CAR:Bangui | AF1 | 4.4016556 N 18.549775 E |
| 07156RCA_2006_466 | KT119659 | 12-May-2006 | Dog | CAR:Bangui | AF1 | 4.3243833 N 18.5261667 E |
| 07157RCA_2006_551 | KT119660 | 19-Jun-2006 | Dog | CAR:Bangui | AF1 | 4.32555 N 18.5261667 E |
| 07158RCA_2006_608 | KT119661 | 20-Jul-2006 | Dog | CAR:Bangui | AF1 | 4.3271833 N 18.5250333 E |
| 07159RCA_2006_792 | KT119662 | 10-Aug-2006 | Dog | CAR:Bangui | AF1 | 4.4081028 N 18.5685056 E |
| 08261RCA_2006_362 | KT119663 | 10-Aug-2006 | Dog | CAR:Bangui | AF1 | 4.3647667 N 18.5438167 E |
| othercity_08262RCA_2006_608 | KT119664 | 16-Oct-2006 | Dog | CAR:Berberati | AF1 |  |
| 07160RCA_2007_030 | KT119665 | 11-Jan-2007 | Dog | CAR:Bangui | AF1 | 4.4238583 N 18.5512556 E |
| 10008RCA_2007_082 | KT119666 | 31-Jan-2007 | Dog | CAR:Bangui | AF1 | 4.3290833 N 18.5346611 E |
| 08264RCA_2007_101 | KT119667 | 06-Feb-2007 | Dog | CAR:Bangui | AF1 | 4.3410833 N 18.5323333 E |
| 07163RCA_2007_164 | KT119668 | 01-Mar-2007 | Dog | CAR:Bangui | AF1 | 4.3871417 N 18.5593778 E |
| 07164RCA_2007_197 | KT119669 | 13-Mar-2007 | Dog | CAR:Bangui | AF1 | 4.3603889 N 18.5460333 E |
| 07165RCA_2007_236 | KT119670 | 27-Mar-2007 | Dog | CAR:Bangui | AF1 | 4.4199 N 18.5616 E |
| 07166RCA_2007_266 | KT119671 | 07-Apr-2007 | Dog | CAR:Bangui | AF1 | 4.4081 N 18.5568667 E |
| 07168RCA_2007_274 | KT119672 | 10-Apr-2007 | Dog | CAR:Bangui | AF1 | 4.3735833 N 18.5568667 E |
| 07169RCA_2007_277 | KT119673 | 11-Apr-2007 | Caprine | CAR:Bangui | AF1 | 4.3389833 N 18.5323667 E |
| 08265RCA_2007_285 | KT119674 | 16-Apr-2007 | Dog | CAR:Bangui | AF1 | 4.37805 N 18.5740167 E |
| 07170RCA_2007_296 | KT119675 | 18-Apr-2007 | Dog | CAR:Bangui | AF1 | 4.33525 N 18.52645 E |
| 07171RCA_2007_312 | KT119676 | 24-Apr-2007 | Dog | CAR:Bangui | AF1 | 4.41245 N 18.5582667 E |
| 07172RCA_2007_312 | KT119677 | 24-Apr-2007 | Dog | CAR:Bangui | AF1 | 4.37715 N 18.6037833 E |
| 07173RCA_2007_337 | KT119678 | 03-May-2007 | Dog | CAR:Bangui | AF1 | 4.3959667 N 18.5502 E |
| 07174RCA_2007_340 | KT119679 | 04-May-2007 | Dog | CAR:Bangui | AF1 | 4.3714 N 18.5256 E |
| 07175RCA_2007_348 | KT119680 | 07-May-2007 | Dog | CAR:Bangui | AF1 | 4.3627 N 18.57045 E |
| 07176RCA_2007_370 | KT119681 | 14-May-2007 | Dog | CAR:Bangui | AF1 | 4.3671333 N 18.5729667 E |
| 07177RCA_2007_373 | KT119682 | 16-May-2007 | Dog | CAR:Bangui | AF1 | 4.4130667 N 18.56365 E |
| 07180RCA_2007_425 | KT119683 | 04-Jun-2007 | Dog | CAR:Bangui | AF1 | 4.41175 N 18.4720833 E |
| 07181RCA_2007_446 | KT119684 | 12-Jun-2007 | Dog | CAR:Bangui | AF1 | 4.3887667 N 18.5433333 E |
| 07182RCA_2007_452 | KT119685 | 14-Jun-2007 | Dog | CAR:Bangui | AF1 | 4.3951667 N 18.54425 E |
| 07183RCA_2007_455 | KT119686 | 15-Jun-2007 | Dog | CAR:Bangui | AF1 | 4.3973 N 18.5391667 E |
| 07184RCA_2007_471 | KT119687 | 21-Jun-2007 | Dog | CAR:Bangui | AF1 | 4.3747333 N 18.5686 E |
| 07185RCA_2007_512 | KT119688 | 06-Jul-2007 | Cat | CAR:Bangui | AF1 | 4.3573333 N 18.5575333 E |
| 07186RCA_2007_533 | KT119689 | 21-Jul-2007 | Dog | CAR:Bangui | AF1 | 4.3453833 N 18.54425 E |
| 07187RCA_2007_559 | KT119690 | 23-Jul-2007 | Dog | CAR:Bangui | AF1 | 4.3525833 N 18.5310333 E |
| 07188RCA_2007_567 | KT119691 | 26-Jul-2007 | Dog | CAR:Bangui | AF1 | 4.3618333 N 18.53515 E |
| 07189RCA_2007_589 | KT119692 | 03-Aug-2007 | Dog | CAR:Bangui | AF1 | 4.3990417 N 18.5659583 E |
| 07190RCA_2007_603 | KT119693 | 08-Aug-2007 | Dog | CAR:Bangui | AF1 | 4.4107333 N 18.5005 E |
| 07191RCA_2007_608 | KT119694 | 10-Aug-2007 | Dog | CAR:Bangui | AF1 | 4.3712667 N 18.6100333 E |
| 07192RCA_2007_608 | KT119695 | 10-Aug-2007 | Dog | CAR:Bangui | AF1 | 4.3772 N 18.5470833 E |
| 07193RCA_2007_625 | KT119696 | 16-Aug-2007 | Dog | CAR:Bangui | AF1 | 4.4126361 N 18.5409417 E |
| 07194RCA_2007_636 | KT119697 | 20-Aug-2007 | Dog | CAR:Bangui | AF1 | 4.3952389 N 18.564975 E |
| 07195RCA_2007_655 | KT119698 | 27-Aug-2007 | Dog | CAR:Bangui | AF1 | 4.4249556 N 18.535975 E |
| 10009RCA_2007_701 | KT119699 | 13-Sep-2007 | Dog | CAR:Bangui | AF1 | 4.3536333 N 18.5405667 E |
| 10010RCA_2007_701 | KT119700 | 13-Sep-2007 | Dog | CAR:Bangui | AF1 | 4.4211417 N 18.5397806 E |
| 10011RCA_2007_737 | KT119701 | 26-Sep-2007 | Dog | CAR:Bangui | AF1 | 4.4499139 N 18.5354583 E |
| 10012RCA_2007_838 | KT119702 | 02-Nov-2007 | Dog | CAR:Bangui | AF1 | 4.4651028 N 18.530575 E |
| 10013RCA_2007_874 | KT119703 | 15-Nov-2007 | Dog | CAR:Bangui | AF1 | 4.4515139 N 18.533675 E |
| 10014RCA_2007_907 | KT119704 | 27-Nov-2007 | Dog | CAR:Bangui | AF1 | 4.3703333 N 18.56875 E |
| 10016RCA_2007_951 | KT119705 | 13-Dec-2007 | Dog | CAR:Bangui | AF1 | 4.3622167 N 18.5525667 E |
| 10017RCA_2007_951 | KT119706 | 13-Dec-2007 | Dog | CAR:Bangui | AF1 | 4.4044444 N 18.533975 E |
| 10018RCA_2007_962 | KT119707 | 17-Dec-2007 | Dog | CAR:Bangui | AF1 | 4.4601417 N 18.520825 E |
| 10019RCA_2007_962 | KT119708 | 17-Dec-2007 | Dog | CAR:Bangui | AF1 | 4.3983528 N 18.559925 E |
| 10020RCA_2007_981 | KT119709 | 24-Dec-2007 | Dog | CAR:Bangui | AF1 | 4.3582333 N 18.51855 E |
| othercity_08267RCA_2008_027 | KT119710 | 10-Jan-2008 | Dog | CAR:Carnot | AF1 |  |
| 08268RCA_2008_109 | KT119711 | 09-Feb-2008 | Dog | CAR:Bangui | AF1 | 4.3657667 N 18.5234 E |
| 08269RCA_2008_172 | KT119712 | 08-Mar-2008 | Dog | CAR:Bangui | AF1 | 4.3649833 N 18.5635167 E |
| 08271RCA_2008_306 | KT119713 | 21-Apr-2008 | Dog | CAR:Bangui | AF1 | 4.3861 N 18.5430167 E |
| 08272RCA_2008_309 | KT119714 | 22-Apr-2008 | Dog | CAR:Bangui | AF1 | 4.4557 N 18.53235 E |
| 08273RCA_2008_426 | KT119715 | 04-Jun-2008 | Caprine | CAR:Bangui | AF1 | 4.4418194 N 18.5311056 E |
| 08275RCA_2008_462 | KT119716 | 17-Jun-2008 | Dog | CAR:Bangui | AF1 | 4.4219167 N 18.5399 E |
| 08277RCA_2008_503 | KT119717 | 02-Jul-2008 | Dog | CAR:Bangui | AF1 | 4.4101667 N 18.5328583 E |
| 08278RCA_2008_505 | KT119718 | 03-Jul-2008 | Dog | CAR:Bangui | AF1 | 4.45675 N 18.5294167 E |
| 08281RCA_2008_546 | KT119719 | 18-Jul-2008 | Dog | CAR:Bangui | AF1 | 4.3912 N 18.5403 E |
| 08282RCA_2008_579 | KT119720 | 30-Jul-2008 | Dog | CAR:Bangui | AF1 | 4.4080667 N 18.5381833 E |
| 08283RCA_2008_596 | KT119721 | 05-Aug-2008 | Dog | CAR:Bangui | AF1 | 4.4240667 N 18.53715 E |
| 08284RCA_2008_637 | KT119722 | 20-Aug-2008 | Dog | CAR:Bangui | AF1 | 4.4053167 N 18.5342333 E |
| 08285RCA_2008_650 | KT119723 | 25-Aug-2008 | Dog | CAR:Bangui | AF1 | 4.36385 N 18.5467 E |
| 08286RCA_2008_688 | KT119724 | 08-Sep-2008 | Dog | CAR:Bangui | AF1 | 4.3577667 N 18.5406 E |
| 08287RCA_2008_735 | KT119725 | 25-Sep-2008 | Dog | CAR:Bangui | AF1 | 4.3732333 N 18.5168 E |
| 08290RCA_2008_888 | KT119726 | 20-Nov-2008 | Dog | CAR:Bangui | AF1 | 4.36305 N 18.5649 E |
| 08291RCA_2008_908 | KT119727 | 26-Nov-2008 | Dog | CAR:Bangui | AF1 | 4.3649667 N 18.55335 E |
| othercity_08292RCA_2008_907 | KT119728 | 27-Nov-2008 | dog | CAR:Bakongo | AF1 |  |
| 08293RCA_2008_929 | KT119729 | 05-Dec-2008 | Dog | CAR:Bangui | AF1 | 4.36295 N 18.5190333 E |
| 08294RCA_2008_934 | KT119730 | 07-Dec-2008 | Dog | CAR:Bangui | AF1 | 4.3418667 N 18.5433833 E |
| 08295RCA_2008_994 | KT119731 | 29-Dec-2008 | Dog | CAR:Bangui | AF1 | 4.3735167 N 18.5507333 E |
| 08296RCA_2008_997 | KT119732 | 30-Dec-2008 | Dog | CAR:Bangui | AF1 | 4.4345333 N 18.5392167 E |
| 09037RCA_2009_096 | KT119733 | 04-Feb-2009 | Dog | CAR:Bangui | AF1 | 4.3301 N 18.51845 E |
| 09038RCA_2009_115 | KT119734 | 11-Feb-2009 | Dog | CAR:Bangui | AF1 | 4.4042333 N 18.5561 E |
| 09039RCA_2009_134 | KT119735 | 18-Feb-2009 | Dog | CAR:Bangui | AF1 | 4.433 N 18.5462167 E |
| 09041RCA_2009_266 | KT119736 | 07-Apr-2009 | Dog | CAR:Bangui | AF1 | 4.3673 N 18.5859667 E |
| 09044RCA_2009_342 | KT119737 | 05-May-2009 | Dog | CAR:Bangui | AF1 | 4.3736 N 18.5585833 E |
| 09045RCA_2009_367 | KT119738 | 14-May-2009 | Dog | CAR:Bangui | AF1 | 4.3925167 N 18.55155 E |
| 09046RCA_2009_403 | KT119739 | 27-May-2009 | Dog | CAR:Bangui | AF1 | 4.3749167 N 18.5491667 E |
| 09047RCA_2009_408 | KT119740 | 29-May-2009 | Dog | CAR:Bangui | AF1 | 4.3747167 N 18.5745833 E |
| 09048RCA_2009_436 | KT119741 | 08-Jun-2009 | Cat | CAR:Bangui | AF1 | 4.3683833 N 18.5711667 E |
| 09049RCA_2009_438 | KT119742 | 09-May-2009 | Dog | CAR:Bangui | AF1 | 4.4043333 N 18.5395 E |
| 09050RCA_2009_460 | KT119743 | 17-Jun-2009 | Dog | CAR:Bangui | AF1 | 4.3862667 N 18.5535833 E |
| 09051RCA_2009_474 | KT119744 | 22-Jun-2009 | Dog | CAR:Bangui | AF1 | 4.3796167 N 18.5554 E |
| 09052RCA_2009_479 | KT119745 | 24-Jun-2009 | Dog | CAR:Bangui | AF1 | 4.37135 N 18.5698167 E |
| 09055RCA_2009_627 | KT119746 | 09-Aug-2009 | Dog | CAR:Bangui | AF1 | 4.3698833 N 18.5718833 E |
| 09056RCA_2009_627 | KT119747 | 17-Aug-2009 | Caprine | CAR:Bangui | AF1 | 4.38965 N 18.5484333 E |
| 09060RCA_2009_945 | KT119748 | 11-Dec-2009 | Dog | CAR:Bangui | AF1 | 4.39255 N 18.5378833 E |
| 10005RCA_2010_118 | KT119749 | 12-Feb-2010 | Dog | CAR:Bangui | AF1 | 4.4571 N 18.5510833 E |
| 10007RCA_2010_238 | KT119750 | 28-Mar-2010 | Dog | CAR:Bangui | AF1 | 4.3608667 N 18.5768833 E |
| 10021RCA_2010_548 | KT119751 | 19-Jul-2010 | Dog | CAR:Bangui | AF1 | 4.3838 N 18.5548833 E |
| 10022RCA_2010_970 | KT119752 | 20-Dec-2010 | Dog | CAR:Bangui | AF1 | 4.45315 N 18.5334667 E |
| othercity_11032RCA_2011_559 | KT119753 | 23-Jun-2011 | Dog | CAR:Yaloke | AF1 |  |
| othercity_07149RCA_2004_628 | KT119754 | 17-Aug-2004 | Dog | CAR:Bossangoa | AF2 |  |
| othercity_07151RCA_2004_708 | KT119755 | 15-Sep-2004 | Dog | CAR:Bossangoa | AF2 |  |
| 10015RCA_2007_929 | KT119756 | 05-Dec-2007 | Dog | CAR:Bangui | AF2 | 4.4073 N 18.5614167 E |
| 08266RCA_2008_036 | KT119757 | 13-Jan-2008 | Dog | CAR:Bangui | AF2 | 4.3638833 N 18.6185833 E |
| 08270RCA_2008_238 | KT119758 | 27-Mar-2008 | Dog | CAR:Bangui | AF2 | 4.4056N 18.5573 E |
| 08274RCA_2008_432 | KT119759 | 06-Jun-2008 | Dog | CAR:Bangui | AF2 | 4.4011 N 18.5786 E |
| 08276RCA_2008_464 | KT119760 | 18-Jun-2008 | Dog | CAR:Bangui | AF2 | 4.34 N 18.5344833 E |
| 08280RCA_2008_533 | KT119761 | 14-Jul-2008 | Caprine | CAR:Bangui | AF2 | 4.4062667 N 18.57935 E |
| 08288RCA_2008_806 | KT119762 | 21-Oct-2008 | Dog | CAR:Bangui | AF2 | 4.3536333 N 18.5421333 E |
| othercity_08289RCA_2008_814 | KT119763 | 24-Oct-2008 | Dog | CAR:Kaga Bandoro | AF2 |  |
| othercity_09036RCA_2009_027 | KT119764 | 10-Jan-2009 | Dog | CAR:Kabo Ouham | AF2 |  |
| othercity_09040RCA_2009_200 | KT119765 | 14-Mar-2009 | Dog | CAR:Bouca Ouham | AF2 |  |
| othercity_09042RCA_2009_266 | KT119766 | 07-Apr-2009 | Dog | CAR:Kabo Ouham | AF2 |  |
| othercity_09057RCA_2009_674 | KT119767 | 03-Sep-2009 | Dog | CAR:Markounda Ouham | AF2 |  |
| 12001RCA_2012_036 | KT119768 | 13-Jan-2012 | Dog | CAR:Bangui | AF2 | 4.4013917 N 18.5652417 E |
| othercity_12002RCA_2012_167 | KT119769 | 01-Mar-2012 | Dog | CAR:Sibut | AF2 |  |
| 12003RCA_2012_180 | KT119770 | 06-Mar-2012 | Dog | CAR:Bangui | AF2 | 4.3756194 N 18.6163667 E |
| 8693GAB_1986_211 | KT119771 | 20-Mar-1986 | Dog | GABON:Libreville | AF1 |  |
| 8698GAB_1986_392 | KT119772 | 26-May-1986 | Dog | GABON:Libreville | AF1 |  |
| 8801CAM_1987_573 | KT119773 | 28-Jul-1987 | Dog | CAMEROUN:Bibemi | AF2 |  |
| 8802CAM_1987_800 | KT119774 | 19-Oct-1987 | Dog | CAMEROUN:Toubouro | AF2 |  |
| 8907GAB_1989_123 | KT119775 | 15-Feb-1989 | Dog | GABON:Port Gentil | AF1 |  |
| 8915ZAI_1989_247 | KT119776 | 17-May-1989 | Dog | REPUBLIQUE DEMOCRATIQUE DU CONGO:Kinshasa | AF1 |  |
| 8914GAB_1989_458 | KT119777 | 19-Jun-1989 | Dog | GABON:Port Gentil | AF1 |  |
| 9021TCH_1990_507 | KT119778 | 06-Jul-1990 | Dog | CHAD:N'djamena | AF2 |  |
| 9218TCH_1992_322 | KT119779 | 04-May-1992 | Dog | CHAD:Moundou | AF2 |  |
| 9228CAF_1992_336 | KT119780 | 02-May-1992 | Dog | CAR:Bangui | AF1 |  |
| 9229CAF_1992_541 | KT119781 | 16-Jul-1992 | Dog | CAR:Bangui | AF1 |  |
| 9236CAM_1992_828 | KT119782 | 30-Oct-1992 | Cat | CAMEROUN:Yaounde | AF1 |  |
| 9350GAB_1993_679 | KT119783 | 11-Sep-1993 | Dog | GABON:Libreville | AF1 |  |
| 9502CAM_1995_912 | KT119784 | 29-Nov-1994 | Dog | CAMEROUN:Yaya | AF2 |  |
| 9608GAB_1996_240 | KT119785 | 28-Mar-1996 | Dog | GABON:Libreville | AF1 |  |
| 97138TCH_1997_923 | KT119786 | 03-Dec-1997 | Dog | CHAD:N’djamena | AF2 |  |
| 11018CAM_2009_079 | KT119787 | 29-Jan-2010 | Dog | CAMEROUN:Soa | AF1 |  |
| 11019CAM_2009_378 | KT119788 | 18-May-2010 | Dog | CAMEROUN:Yaounde | AF1 |  |
| 11020CAM_2009_868 | KT119789 | 13-Nov-2009 | Dog | CAMEROUN:Yaounde | AF1 |  |
| 11022CAM_2010_989 | KT119790 | 27-Dec-2010 | Dog | CAMEROUN:Obala | AF1 |  |
| 11023CAM_2010_855 | KT119791 | 08-May-2011 | Dog | CAMEROUN:Obala | AF1 |  |
| 11025CAM_2010_871 | KT119792 | 14-Nov-2009 | Dog | CAMEROUN:Lekie | AF1 |  |
| 11027CAM_2010_690 | KT119793 | 07-Sep-2010 | Dog | CAMEROUN:Yaounde | AF1 |  |
| 11028CAM_2011_866 | KT119794 | 12-Nov-2009 | Dog | CAMEROUN:Yaounde | AF1 |  |
| 11029CAM_2011_090 | KT119795 | 02-Feb-2011 | Dog | CAMEROUN:Yaounde | AF1 |  |

Text S1: Bayesian evolutionary analysis of spatiotemporal RABV dynamics.

The posterior set of phylogenetic trees from BEAST was used to identify separate introductions of RABV to Bangui. Let *L* be a length of time, in years, long enough that rabies being transmitted down a single chain while the population of infected dogs in the city was being sampled (with probability less than 1) upon death, we would expect to take at least one sample from a dog in the chain. Let *P* be a conservative estimate of the time from infection to death of an infected dog. If, initially, we assume that we have a correct time-scaled phylogeny *T* for the set of sequences, we suggest that it is reasonable to assume that a node *a* of *T* represents the earliest node of a new viral introduction to Bangui if the following are true:

1. *a,* or at least one descendant of *a*, is a tip corresponding to a Bangui isolate
2. Sampling was occurring at the time *t_a_* of *a*, and was occurring for at least *L* years before *t_a_.*
3. There is no node *b* that is an ancestor of *a*, occurs less than *L* years before *t_a_*, and has a descendant tip *c* that is not descended from *a*, corresponds to an isolate sampled from Bangui, and occurs less than *P* years after *b*.
4. *a* has no ancestor *d*, occurring less than *P* years before *t_a_*, for which the previous three conditions are also true.

If condition 3 were not true, the virus at *b* could have infected the dog sampled at *c*, so another dog in a Bangui transmission chain including the dog infected at *a* may have been sampled. If condition 4 were not true, then the virus at *a* could have infected the same dog as the virus at *d*, so the latter would represent the genuine introduction. Moving from a single phylogeny to a sample from the posterior distribution of phylogenies, we considered a set of isolates to be descended from such a new introduction if they formed a clade with at least 95% posterior probability, and that there was also at least 95% posterior probability that the above conditions were met for the root node of that clade. As described in the Results section of the main text, the two waves of AF1 sequences from Bangui and the AF2 sequences were always treated as at least three separate introductions due the inability of this procedure to separate them for larger values of *L* due to the lack of sampling prior to 2003.

**Table B: Summary of the fit for models where $R_{t}$ varied monthly while $\lambda_{t}$ was either kept constant varied every 3 months or yearly.**

|  | mean deviance | Estimated parameters | | | |
| --- | --- | --- | --- | --- | --- |
|  |  | $R_{t}$ related | | $\lambda_{t}$ related | |
|  |  | estimation | 95%CrI | estimation | 95%CrI |
| $\lambda_{t}$ constant | 1317 | $\sigma_{R_{t}}^{2}=0.023$ $\sigma_{R_{t}epi}^{2}=0.103$* | $\left[0.001-0.099\right]$ $\left[0.002-0.292\right]$ | $\lambda_{t}=0.13$ | $\left[0.07-0.27\right]$ |
|  |  | Median $R_{t}$ ranged within $\left[0.76-1.32\right]$ | |  | |
| Quarterly $\lambda_{t}$ variation | 1360 | $\sigma_{R_{t}}^{2}=0.040$ $\sigma_{R_{t}epi}^{2}=0.135$* | $\left[0.003-0.137\right]$ $\left[0.007-0.430\right]$ | $\sigma_{\lambda_{t}}^{2}=0.261$ | $\left[0.012-0.578\right]$ |
|  |  | Median $R_{t}$ ranged within $\left[0.41-1.23\right]$ | | Median $\lambda_{t}$ ranged within $\left[0.0-1.1\right]$ | |
| Yearly $\lambda_{t}$ variation | 1322 | $\sigma_{R_{t}}^{2}=0.022$ $\sigma_{R_{t}epi}^{2}=0.141$* | $\left[0.005-0.104\right]$ $\left[0.024-0.338\right]$ | $\sigma_{\lambda_{t}}^{2}=0.239$ | $\left[0.009-0.773\right]$ |
|  |  | Median $R_{t}$ ranged within $\left[0.65-1.25\right]$ | | Median $\lambda_{t}$ ranged within $\left[0.01-0.53\right]$ | |

*: $\sigma_{R_{t}epi}^{2}$ represents the increased volatility in $R_{t}$ during the 3 larger outbreaks. Therefore, during a year in 1999, 2001 and 2008, $R_{t}$ was allowed to vary more rapidly.

**Table C: Sensitivity analysis on the assumption on the surveillance effort $\rho$.**

The sensitivity analysis was performed by running the model with monthly $R_{t}$ variation and constant $\lambda_{t}$ variation.

|  | mean deviance | Estimated parameters | | | |
| --- | --- | --- | --- | --- | --- |
|  |  | $R_{t}$ related | | $\lambda_{t}$ related | |
| $\rho$ |  | estimation | 95%CrI | Estimation | 95%CrI |
| 20% | 1317 | $\sigma_{R_{t}}^{2}=0.023$ $\sigma_{R_{t}epi}^{2}=0.103$* | $\left[0.001-0.099\right]$ $\left[0.002-0.292\right]$ | $\lambda_{t}=0.13$ | $\left[0.07-0.27\right]$ |
|  |  | Median $R_{t}$ ranged within $\left[0.76-1.32\right]$ | |  | |
| 5% | 1321 | $\sigma_{R_{t}}^{2}=0.038$ $\sigma_{R_{t}epi}^{2}=0.166$* | $\left[0.006-0.139\right]$ $\left[0.011-0.377\right]$ | $\lambda_{t}=0.33$ | $\left[0.11-1.08\right]$ |
|  |  | Median $R_{t}$ ranged within $\left[0.72-1.53\right]$ | |  | |
| 10% | 1319 | $\sigma_{R_{t}}^{2}=0.032$ $\sigma_{R_{t}epi}^{2}=0.137$* | $\left[0.003-0.150\right]$ $\left[0.004-0.353\right]$ | $\lambda_{t}=0.20$ | $\left[0.08-0.59\right]$ |
|  |  | Median $R_{t}$ ranged within $\left[0.79-1.46\right]$ | |  | |
| 50% | 1321 | $\sigma_{R_{t}}^{2}=0.026$ $\sigma_{R_{t}epi}^{2}=0.020$* | $\left[0.001-0.111\right]$ $\left[0.025-0.189\right]$ | $\lambda_{t}=0.09$ | $\left[0.05-0.14\right]$ |
|  |  | Median $R_{t}$ ranged within $\left[0.72-1.01\right]$ | |  | |

*: $\sigma_{R_{t}epi}^{2}$ represents the increased volatility in $R_{t}$ during the 3 larger outbreaks. Therefore, during a year in 1999, 2001 and 2008, $R_{t}$ was allowed to vary more rapidly.

**Table D: List of primers used in this study**

| Gene | Full Name | Primer Sequence 5'-3' |
| --- | --- | --- |
| Nucleoprotein | N127 S | ATGTAACACCTCTACAATGG |
|  | N570 AS | AGTTGTCATTAGGGTATGGT |
|  | N668 S | GCAATCAGAGTGGGCACAGT |
|  | N904 S | AAGAATGTTCGAGCCAGGGCAG |
|  | N1280 S | AGTCAGTTCTAATCATCAAGC |
|  | N1256 AS | GATCTCTTTAGTCGACCTCC |
|  | N1596 AS | ATCTCAAGATCAGCCAGACCGGC |
| Phosphoprotein | P1744 S | GTTGGAGTCCAAATAGTCAG |
|  | P1969 S | ATCGACCCAAACCACTGGTCGAG |
|  | P2070 AS | AGCTTTCAGCAATCTGGTGA |
|  | P2316 AS | ATCATCTTGCATGAYTTTRTTTA |
|  | P2417 S | GCAACACCACTGATAAAATG |
|  | P2597 AS | CGGAGGGGCTGATACAGGAGAGG |
| Matrice | M2967 S | GTCAACTATGGTCTGACATG |
|  | M2986 AS | CATGTCAGACCATAGTTGAC |
| Glycoprotein | G3407 S | ACGAAGGATGCACCAACCT |
|  | G3668 AS | TAGTCAGGGTACGGATTGT |
|  | G3846 S | CTCGAGGGTCTTCCCTAGCGGG |
|  | G4176 S | TCCATCATGACCACCAAGTC |
|  | G4197 AS | CTGACTTGGTGGTCATGATG |
|  | G4684 AS | CCCCCGAGACCTGTTTGTGCAC |
|  | G4783 S | ATATCTTCATGGGARTCRTA |
|  | G4871 AS | TGATCTTCGGAACTTGAAGC |
| G-L intergenic region | PSI5264 AS | CCAGCCCTTTATAATTATGC |

**Figure A**: Detailed figure showing the MCC tree of 162 sequences from the Central African Republic and other locations in Africa estimated from 5000 nt of dog RABV genome with names of the tips. Tips representing isolates from Bangui are coloured according to the selected subtypes of RABV; other tips are colored by location. Tip times are scaled to the date of sampling (years) and branches are estimated in time units as indicated by the time bar. Posterior clade probability values (>0.9) are shown for key nodes

**
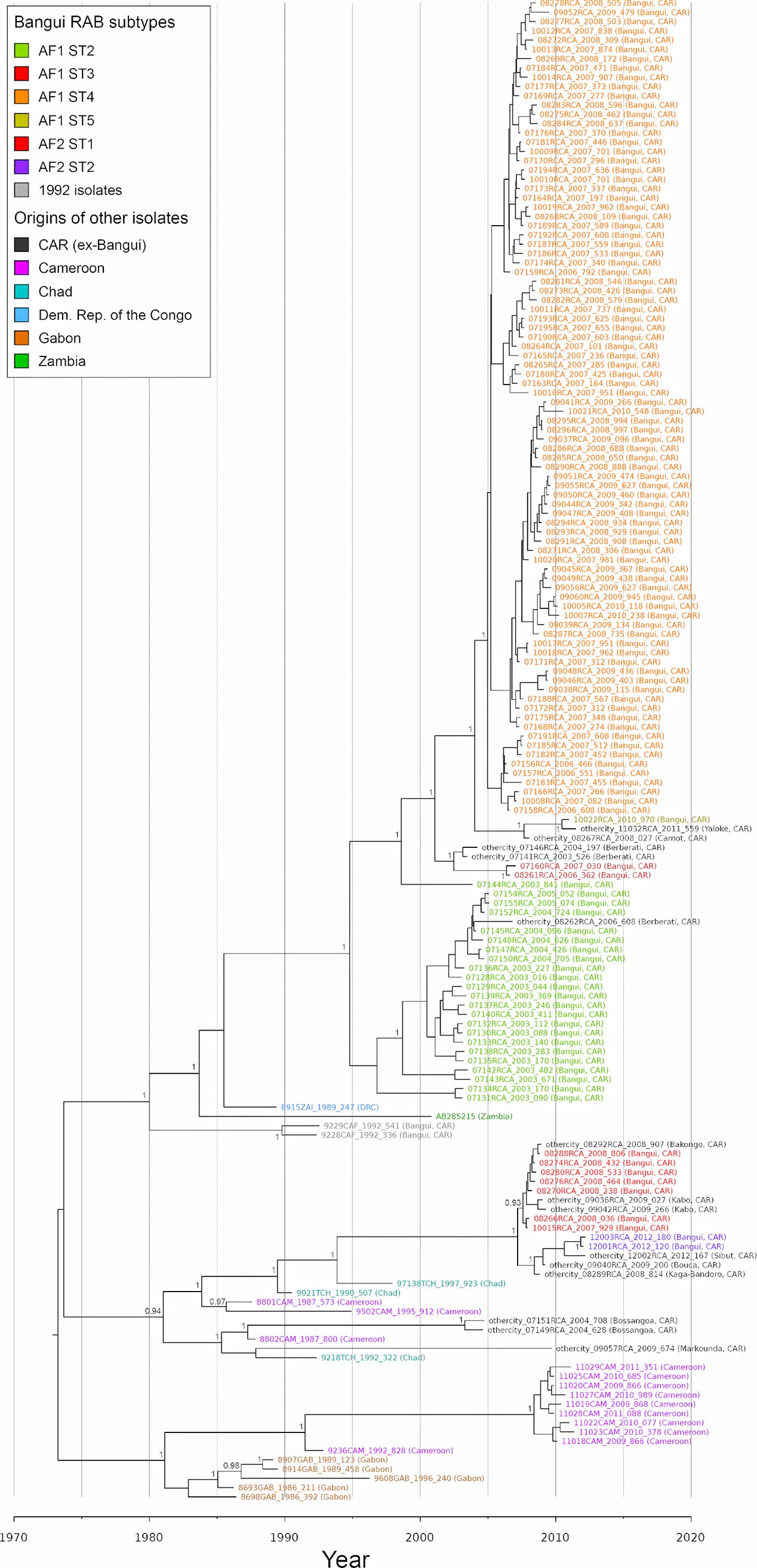
**

**Figure B: Diffusion of RABV in Bangui.**

**Figure B1**: 80% highest posterior density (HPD) regions (red areas) for areas of Bangui where the AF1 ST4 strain of RABV was present, at different times during the epidemic, taken from the phylogeographical analysis. One slice per 6 months is provided for the period from January 2006 (A) to January 2010 (I). Maps were generated using Map images © OpenStreetMap contributors (<http://www.openstreetmap.org>). Map data i available under the Open Database Licence and map tiles under the Creative Commons Attribution-ShareAlike 2.0 licence (see <http://www.openstreetmap.org/copyright>).

**Figure B2:** The maximum clade credibility tree for the ST1 AF4 RABV sequences (2007-2010), superimposed on a map of Bangui. Blue circles represent tips and are positioned at the location at which the corresponding viral isolate was sampled; internal nodes are positioned as reconstructed by the continuous diffusion model.  The map background was generated using Map images © OpenStreetMap contributors (<http://www.openstreetmap.org>). Map data is available under the Open Database Licence and map tiles under the Creative Commons Attribution-ShareAlike 2.0 licence (see <http://www.openstreetmap.org/copyright>).


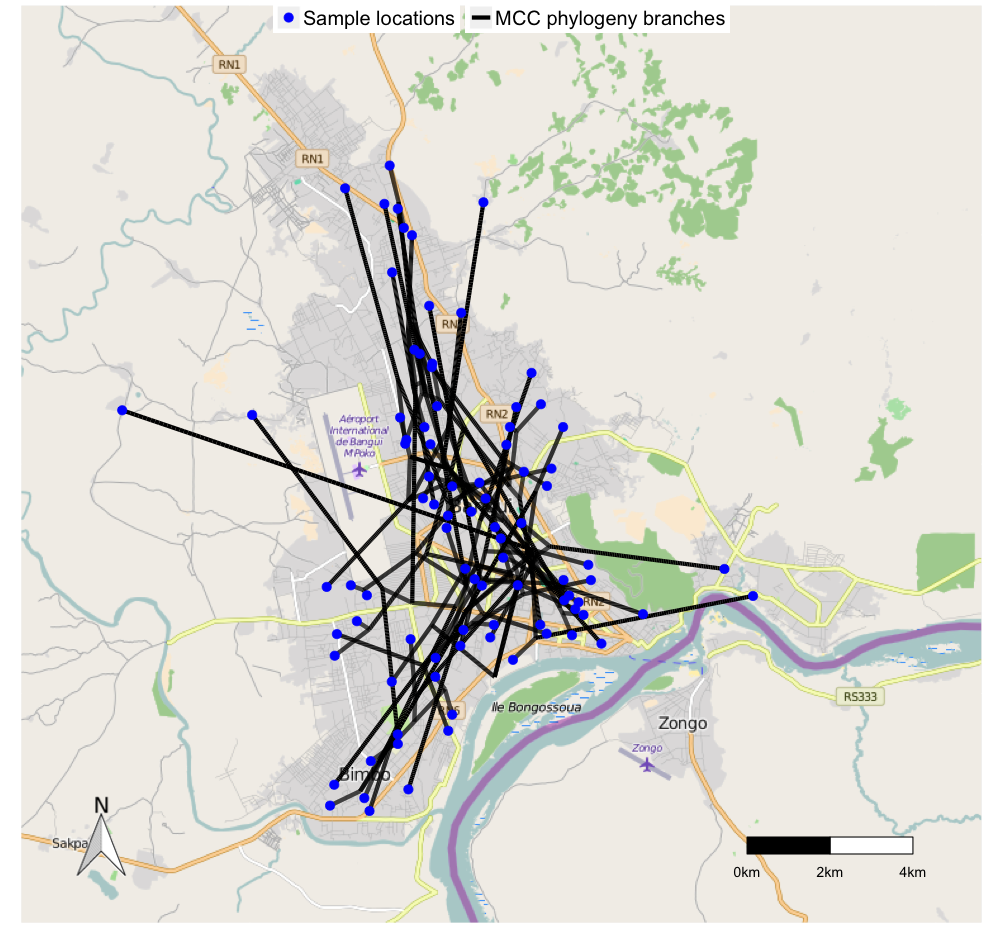


**Figure C: Fitting results with quarterly variation of $\lambda_{t}$.**


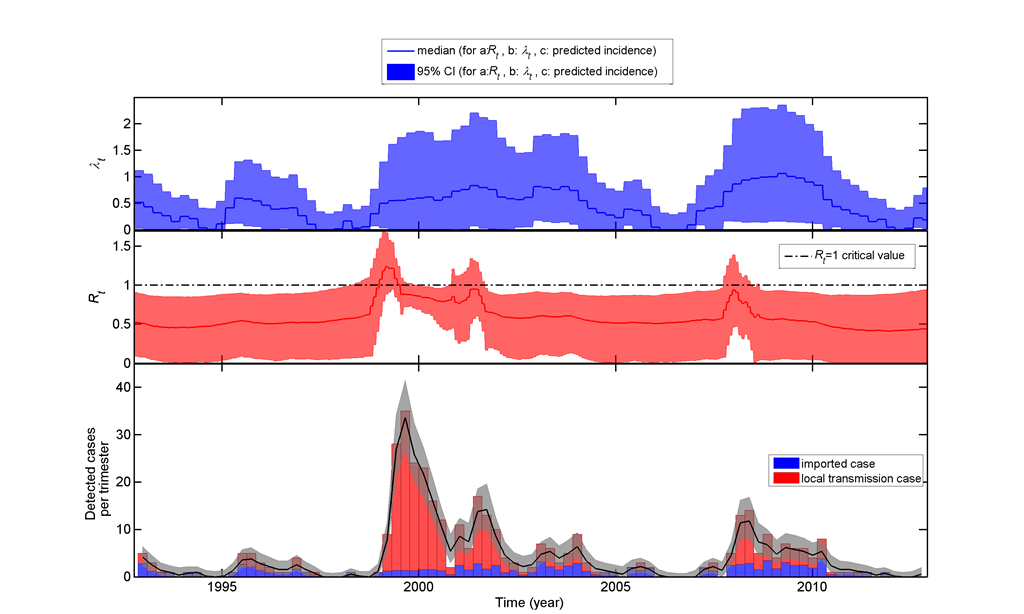


Estimation of (a) the rate of introduction of rabid dogs in Bangui, $\lambda_{t}$ (in rabid dog imported to Bangui per week), (b) the instantaneous effective reproduction number,$R_{t}$, and (c) observed number of rabid dogs infected locally (black bar) or from outside the city (grey bar) and simulated number of rabid dogs from the model (black line: posterior median; grey area: 95% CrI). If $R_{t}$ is above 1, a local self-sustaining epidemic in dogs may occur in the city.

**Figure D: Fitting results with yearly variation of $\lambda_{t}$.**


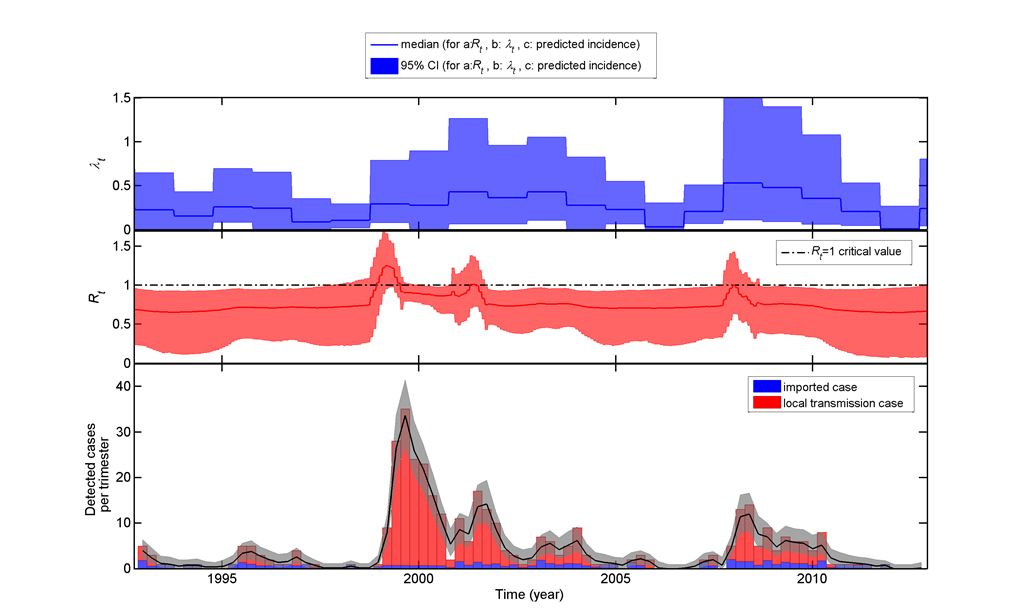


Estimation of (a) the rate of introduction of rabid dogs in Bangui, $\lambda_{t}$ (in rabid dog imported to Bangui per week), (b) the instantaneous effective reproduction number,$R_{t}$, and (c) observed number of rabid dogs infected locally (black bar) or from outside the city (grey bar) and simulated number of rabid dogs from the model (black line: posterior median; grey area: 95% CrI). If $R_{t}$ is above 1, a local self-sustaining epidemic in dogs may occur in the city.

**Figure E: Fitting results with constant importation $\lambda_{t}$.**


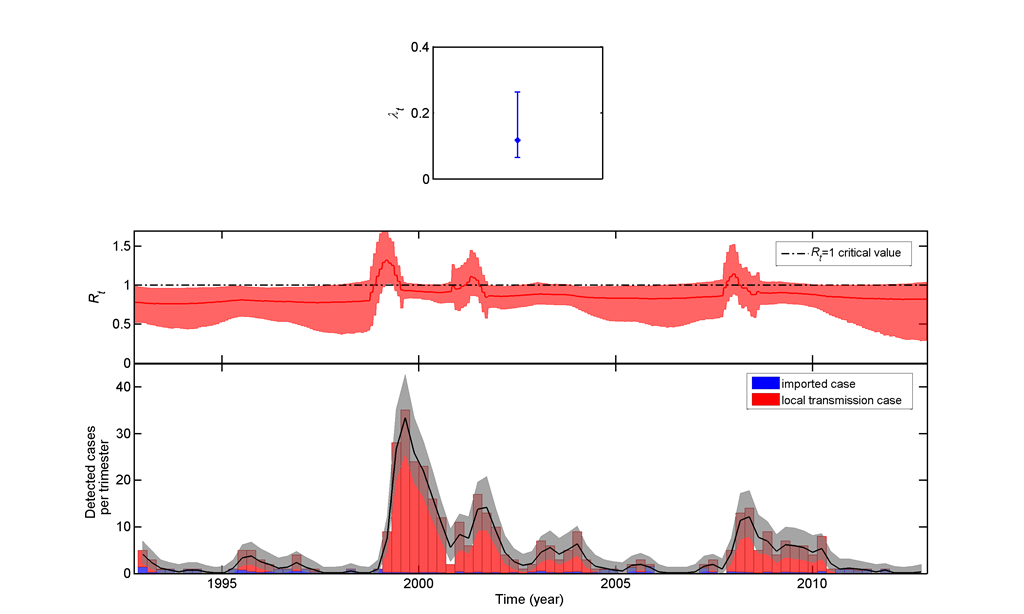


Estimation of (a) the rate of introduction of rabid dogs in Bangui, $\lambda_{t}$ (in rabid dog imported to Bangui per week), (b) the instantaneous effective reproduction number,$R_{t}$, and (c) observed number of rabid dogs infected locally (black bar) or from outside the city (grey bar) and simulated number of rabid dogs from the model (black line: posterior median; grey area: 95% CrI). If $R_{t}$ is above 1, a local self-sustaining epidemic in dogs may occur in the city.
